# Supplementary material for: HIVprotI: an integrated web based platform for prediction and design of HIV proteins inhibitors
Source: J Cheminform. 2018 Mar 9;10:12. doi: 10.1186/s13321-018-0266-y (PMC5845081; doi:10.1186/s13321-018-0266-y)
Supplement: Supplementary file 2 — Additional file 2. Source code of HIVProtI web server. [file 13321_2018_266_MOESM2_ESM.zip › HIVprotI_Source-code/hivprotI/weka-3-6-6/doc/weka/gui/beans/SerializedModelSaver.html]

SerializedModelSaver


---


|  |  |  |
| --- | --- | --- |
| **Overview** \| **Package** \| **Class** \| **Tree** \| **Deprecated** \| **Index** \| **Help** \| **Weka's home** | |  |
| **PREV CLASS**   **NEXT CLASS** | **FRAMES**    **NO FRAMES**     **All Classes** |  |
| SUMMARY: NESTED \| FIELD \| CONSTR \| METHOD | DETAIL: FIELD \| CONSTR \| METHOD |  |


---


## weka.gui.beans Class SerializedModelSaver

```
java.lang.Object
  java.awt.Component
      java.awt.Container
          javax.swing.JComponent
              javax.swing.JPanel
                  weka.gui.beans.SerializedModelSaver
```

**All Implemented Interfaces:**: java.awt.image.ImageObserver, java.awt.MenuContainer, java.io.Serializable, java.util.EventListener, javax.accessibility.Accessible, EnvironmentHandler, BatchClassifierListener, BatchClustererListener, BeanCommon, IncrementalClassifierListener, Visible

---

``` public class SerializedModelSaver extends javax.swing.JPanel implements BeanCommon, Visible, BatchClassifierListener, IncrementalClassifierListener, BatchClustererListener, EnvironmentHandler, java.io.Serializable ```

A bean that saves serialized models

**Version:**
:   $Revision: 7059 $

**Author:**
:   Mark Hall (mhall{[at]}pentaho{[dot]}org

**See Also:**: Serialized Form

---

**Nested Class Summary**

**Nested classes/interfaces inherited from class javax.swing.JComponent**

`javax.swing.JComponent.AccessibleJComponent`

**Nested classes/interfaces inherited from class java.awt.Component**

`java.awt.Component.BaselineResizeBehavior`


| **Field Summary** | |
| --- | --- |
| `static int` | `BINARY` |
| `static java.lang.String` | `FILE_EXTENSION`             the extension for serialized models (binary Java serialization) |
| `static int` | `KOMLV` |
| `static java.util.ArrayList<Tag>` | `s_fileFormatsAvailable`             Available file formats. |
| `static int` | `XSTREAM` |

**Fields inherited from class javax.swing.JComponent**

`TOOL_TIP_TEXT_KEY, UNDEFINED_CONDITION, WHEN_ANCESTOR_OF_FOCUSED_COMPONENT, WHEN_FOCUSED, WHEN_IN_FOCUSED_WINDOW`

**Fields inherited from class java.awt.Component**

`BOTTOM_ALIGNMENT, CENTER_ALIGNMENT, LEFT_ALIGNMENT, RIGHT_ALIGNMENT, TOP_ALIGNMENT`

**Fields inherited from interface java.awt.image.ImageObserver**

`ABORT, ALLBITS, ERROR, FRAMEBITS, HEIGHT, PROPERTIES, SOMEBITS, WIDTH`


**Constructor Summary**

`SerializedModelSaver()`
  
          Constructor.


| **Method Summary** | |
| --- | --- |
| `void` | `acceptClassifier(BatchClassifierEvent ce)`             Accept and save a batch trained classifier. |
| `void` | `acceptClassifier(IncrementalClassifierEvent ce)`             Accept and save an incrementally trained classifier. |
| `void` | `acceptClusterer(BatchClustererEvent ce)`             Accept and save a batch trained clusterer. |
| `boolean` | `connectionAllowed(java.beans.EventSetDescriptor esd)`             Returns true if, at this time, the object will accept a connection according to the supplied EventSetDescriptor. |
| `boolean` | `connectionAllowed(java.lang.String eventName)`             Returns true if, at this time, the object will accept a connection according to the supplied event name. |
| `void` | `connectionNotification(java.lang.String eventName, java.lang.Object source)`             Notify this object that it has been registered as a listener with a source with respect to the supplied event name. |
| `void` | `disconnectionNotification(java.lang.String eventName, java.lang.Object source)`             Notify this object that it has been deregistered as a listener with a source with respect to the supplied event name. |
| `java.lang.String` | `getCustomName()`             Get the custom (descriptive) name for this bean (if one has been set) |
| `java.io.File` | `getDirectory()`             Get the directory that the model(s) will be saved into |
| `Tag` | `getFileFormat()`             Get the file format to use for saving. |
| `java.lang.String` | `getPrefix()`             Get the prefix to prepend to the model file names. |
| `boolean` | `getUseRelativePath()`             Get whether to use relative paths for the directory. |
| `BeanVisual` | `getVisual()`             Get the visual being used by this data source. |
| `java.lang.String` | `globalInfo()`             Global info for this bean. |
| `boolean` | `isBusy()`             Returns true if. |
| `static void` | `saveBinary(java.io.File saveTo, java.lang.Object model, Instances header)`             Save a model in binary form. |
| `static void` | `saveKOML(java.io.File saveTo, java.lang.Object model, Instances header)`             Save a model in KOML deep object serialized XML form. |
| `static void` | `saveXStream(java.io.File saveTo, java.lang.Object model, Instances header)`             Save a model in XStream deep object serialized XML form. |
| `void` | `setCustomName(java.lang.String name)`             Set a custom (descriptive) name for this bean |
| `void` | `setDirectory(java.io.File d)`             Set the directory that the model(s) will be saved into. |
| `void` | `setEnvironment(Environment env)`             Set environment variables to use. |
| `void` | `setFileFormat(Tag ff)`             Set the file format to use for saving. |
| `void` | `setLog(Logger logger)`             Set a log for this bean. |
| `void` | `setPrefix(java.lang.String p)`             Set the prefix to prepend to the model file names. |
| `void` | `setUseRelativePath(boolean rp)`             Set whether to use relative paths for the directory. |
| `void` | `setVisual(BeanVisual newVisual)`             Set the visual for this data source. |
| `void` | `stop()`             Stop any processing that the bean might be doing. |
| `void` | `useDefaultVisual()`             Use the default images for this bean. |
| `Tag` | `validateFileFormat(Tag ff)`             Validate the file format. |

**Methods inherited from class javax.swing.JPanel**

`getAccessibleContext, getUI, getUIClassID, setUI, updateUI`

**Methods inherited from class javax.swing.JComponent**

`addAncestorListener, addNotify, addVetoableChangeListener, computeVisibleRect, contains, createToolTip, disable, enable, firePropertyChange, firePropertyChange, firePropertyChange, getActionForKeyStroke, getActionMap, getAlignmentX, getAlignmentY, getAncestorListeners, getAutoscrolls, getBaseline, getBaselineResizeBehavior, getBorder, getBounds, getClientProperty, getComponentPopupMenu, getConditionForKeyStroke, getDebugGraphicsOptions, getDefaultLocale, getFontMetrics, getGraphics, getHeight, getInheritsPopupMenu, getInputMap, getInputMap, getInputVerifier, getInsets, getInsets, getListeners, getLocation, getMaximumSize, getMinimumSize, getNextFocusableComponent, getPopupLocation, getPreferredSize, getRegisteredKeyStrokes, getRootPane, getSize, getToolTipLocation, getToolTipText, getToolTipText, getTopLevelAncestor, getTransferHandler, getVerifyInputWhenFocusTarget, getVetoableChangeListeners, getVisibleRect, getWidth, getX, getY, grabFocus, isDoubleBuffered, isLightweightComponent, isManagingFocus, isOpaque, isOptimizedDrawingEnabled, isPaintingForPrint, isPaintingTile, isRequestFocusEnabled, isValidateRoot, paint, paintImmediately, paintImmediately, print, printAll, putClientProperty, registerKeyboardAction, registerKeyboardAction, removeAncestorListener, removeNotify, removeVetoableChangeListener, repaint, repaint, requestDefaultFocus, requestFocus, requestFocus, requestFocusInWindow, resetKeyboardActions, reshape, revalidate, scrollRectToVisible, setActionMap, setAlignmentX, setAlignmentY, setAutoscrolls, setBackground, setBorder, setComponentPopupMenu, setDebugGraphicsOptions, setDefaultLocale, setDoubleBuffered, setEnabled, setFocusTraversalKeys, setFont, setForeground, setInheritsPopupMenu, setInputMap, setInputVerifier, setMaximumSize, setMinimumSize, setNextFocusableComponent, setOpaque, setPreferredSize, setRequestFocusEnabled, setToolTipText, setTransferHandler, setVerifyInputWhenFocusTarget, setVisible, unregisterKeyboardAction, update`

**Methods inherited from class java.awt.Container**

`add, add, add, add, add, addContainerListener, addPropertyChangeListener, addPropertyChangeListener, applyComponentOrientation, areFocusTraversalKeysSet, countComponents, deliverEvent, doLayout, findComponentAt, findComponentAt, getComponent, getComponentAt, getComponentAt, getComponentCount, getComponents, getComponentZOrder, getContainerListeners, getFocusTraversalKeys, getFocusTraversalPolicy, getLayout, getMousePosition, insets, invalidate, isAncestorOf, isFocusCycleRoot, isFocusCycleRoot, isFocusTraversalPolicyProvider, isFocusTraversalPolicySet, layout, list, list, locate, minimumSize, paintComponents, preferredSize, printComponents, remove, remove, removeAll, removeContainerListener, setComponentZOrder, setFocusCycleRoot, setFocusTraversalPolicy, setFocusTraversalPolicyProvider, setLayout, transferFocusBackward, transferFocusDownCycle, validate`

**Methods inherited from class java.awt.Component**

`action, add, addComponentListener, addFocusListener, addHierarchyBoundsListener, addHierarchyListener, addInputMethodListener, addKeyListener, addMouseListener, addMouseMotionListener, addMouseWheelListener, bounds, checkImage, checkImage, contains, createImage, createImage, createVolatileImage, createVolatileImage, dispatchEvent, enable, enableInputMethods, firePropertyChange, firePropertyChange, firePropertyChange, firePropertyChange, firePropertyChange, getBackground, getBounds, getColorModel, getComponentListeners, getComponentOrientation, getCursor, getDropTarget, getFocusCycleRootAncestor, getFocusListeners, getFocusTraversalKeysEnabled, getFont, getForeground, getGraphicsConfiguration, getHierarchyBoundsListeners, getHierarchyListeners, getIgnoreRepaint, getInputContext, getInputMethodListeners, getInputMethodRequests, getKeyListeners, getLocale, getLocation, getLocationOnScreen, getMouseListeners, getMouseMotionListeners, getMousePosition, getMouseWheelListeners, getName, getParent, getPeer, getPropertyChangeListeners, getPropertyChangeListeners, getSize, getToolkit, getTreeLock, gotFocus, handleEvent, hasFocus, hide, imageUpdate, inside, isBackgroundSet, isCursorSet, isDisplayable, isEnabled, isFocusable, isFocusOwner, isFocusTraversable, isFontSet, isForegroundSet, isLightweight, isMaximumSizeSet, isMinimumSizeSet, isPreferredSizeSet, isShowing, isValid, isVisible, keyDown, keyUp, list, list, list, location, lostFocus, mouseDown, mouseDrag, mouseEnter, mouseExit, mouseMove, mouseUp, move, nextFocus, paintAll, postEvent, prepareImage, prepareImage, remove, removeComponentListener, removeFocusListener, removeHierarchyBoundsListener, removeHierarchyListener, removeInputMethodListener, removeKeyListener, removeMouseListener, removeMouseMotionListener, removeMouseWheelListener, removePropertyChangeListener, removePropertyChangeListener, repaint, repaint, repaint, resize, resize, setBounds, setBounds, setComponentOrientation, setCursor, setDropTarget, setFocusable, setFocusTraversalKeysEnabled, setIgnoreRepaint, setLocale, setLocation, setLocation, setName, setSize, setSize, show, show, size, toString, transferFocus, transferFocusUpCycle`

**Methods inherited from class java.lang.Object**

`equals, getClass, hashCode, notify, notifyAll, wait, wait, wait`

**Field Detail**

### BINARY

```
public static final int BINARY
```

**See Also:**: Constant Field Values

---


### KOMLV

```
public static final int KOMLV
```

**See Also:**: Constant Field Values

---


### XSTREAM

```
public static final int XSTREAM
```

**See Also:**: Constant Field Values

---


### FILE\_EXTENSION

```
public static final java.lang.String FILE_EXTENSION
```

:   the extension for serialized models (binary Java serialization)

    **See Also:**: Constant Field Values

---


### s\_fileFormatsAvailable

```
public static java.util.ArrayList<Tag> s_fileFormatsAvailable
```

:   Available file formats. Reflection is used to check if classes
    are available for deep object serialization to XML


**Constructor Detail**

### SerializedModelSaver

```
public SerializedModelSaver()
```

:   Constructor.


**Method Detail**

### setCustomName

```
public void setCustomName(java.lang.String name)
```

:   Set a custom (descriptive) name for this bean

    :   **Specified by:**: `setCustomName` in interface `BeanCommon`
    :   **Parameters:**: `name` - the name to use

---


### getCustomName

```
public java.lang.String getCustomName()
```

:   Get the custom (descriptive) name for this bean (if one has been set)

    :   **Specified by:**: `getCustomName` in interface `BeanCommon`
    :   **Returns:**: the custom name (or the default name)

---


### useDefaultVisual

```
public void useDefaultVisual()
```

:   Use the default images for this bean.

    :   **Specified by:**: `useDefaultVisual` in interface `Visible`

---


### setVisual

```
public void setVisual(BeanVisual newVisual)
```

:   Set the visual for this data source.

    :   **Specified by:**: `setVisual` in interface `Visible`
    :   **Parameters:**: `newVisual` - a `BeanVisual` value

---


### getVisual

```
public BeanVisual getVisual()
```

:   Get the visual being used by this data source.

    :   **Specified by:**: `getVisual` in interface `Visible`
    :   **Returns:**: a `BeanVisual` value

---


### connectionAllowed

```
public boolean connectionAllowed(java.beans.EventSetDescriptor esd)
```

:   Returns true if, at this time,
    the object will accept a connection according to the supplied
    EventSetDescriptor.

    :   **Specified by:**: `connectionAllowed` in interface `BeanCommon`
    :   **Parameters:**: `esd` - the EventSetDescriptor **Returns:**: true if the object will accept a connection

---


### connectionAllowed

```
public boolean connectionAllowed(java.lang.String eventName)
```

:   Returns true if, at this time,
    the object will accept a connection according to the supplied
    event name.

    :   **Specified by:**: `connectionAllowed` in interface `BeanCommon`
    :   **Parameters:**: `eventName` - the event **Returns:**: true if the object will accept a connection

---


### connectionNotification

```
public void connectionNotification(java.lang.String eventName,
                                   java.lang.Object source)
```

:   Notify this object that it has been registered as a listener with
    a source with respect to the supplied event name.

    :   **Specified by:**: `connectionNotification` in interface `BeanCommon`
    :   **Parameters:**: `eventName` - the event: `source` - the source with which this object has been registered as a listener

---


### disconnectionNotification

```
public void disconnectionNotification(java.lang.String eventName,
                                      java.lang.Object source)
```

:   Notify this object that it has been deregistered as a listener with
    a source with respect to the supplied event name.

    :   **Specified by:**: `disconnectionNotification` in interface `BeanCommon`
    :   **Parameters:**: `eventName` - the event: `source` - the source with which this object has been registered as a listener

---


### setLog

```
public void setLog(Logger logger)
```

:   Set a log for this bean.

    :   **Specified by:**: `setLog` in interface `BeanCommon`
    :   **Parameters:**: `logger` - a `weka.gui.Logger` value

---


### stop

```
public void stop()
```

:   Stop any processing that the bean might be doing.

    :   **Specified by:**: `stop` in interface `BeanCommon`

---


### isBusy

```
public boolean isBusy()
```

:   Returns true if. at this time, the bean is busy with some
    (i.e. perhaps a worker thread is performing some calculation).

    :   **Specified by:**: `isBusy` in interface `BeanCommon`
    :   **Returns:**: true if the bean is busy.

---


### acceptClusterer

```
public void acceptClusterer(BatchClustererEvent ce)
```

:   Accept and save a batch trained clusterer.

    :   **Specified by:**: `acceptClusterer` in interface `BatchClustererListener`
    :   **Parameters:**: `ce` - a `BatchClassifierEvent` value

---


### acceptClassifier

```
public void acceptClassifier(IncrementalClassifierEvent ce)
```

:   Accept and save an incrementally trained classifier.

    :   **Specified by:**: `acceptClassifier` in interface `IncrementalClassifierListener`
    :   **Parameters:**: `ce` - the BatchClassifierEvent containing the classifier

---


### acceptClassifier

```
public void acceptClassifier(BatchClassifierEvent ce)
```

:   Accept and save a batch trained classifier.

    :   **Specified by:**: `acceptClassifier` in interface `BatchClassifierListener`
    :   **Parameters:**: `ce` - the BatchClassifierEvent containing the classifier

---


### saveBinary

```
public static void saveBinary(java.io.File saveTo,
                              java.lang.Object model,
                              Instances header)
                       throws java.io.IOException
```

:   Save a model in binary form.

    :   **Parameters:**: `saveTo` - the file name to save to: `model` - the model to save: `header` - the header of the data that was used to train the model (optional) **Throws:**: `java.io.IOException`

---


### saveKOML

```
public static void saveKOML(java.io.File saveTo,
                            java.lang.Object model,
                            Instances header)
                     throws java.lang.Exception
```

:   Save a model in KOML deep object serialized XML form.

    :   **Parameters:**: `saveTo` - the file name to save to: `model` - the model to save: `header` - the header of the data that was used to train the model (optional) **Throws:**: `java.lang.Exception`

---


### saveXStream

```
public static void saveXStream(java.io.File saveTo,
                               java.lang.Object model,
                               Instances header)
                        throws java.lang.Exception
```

:   Save a model in XStream deep object serialized XML form.

    :   **Parameters:**: `saveTo` - the file name to save to: `model` - the model to save: `header` - the header of the data that was used to train the model (optional) **Throws:**: `java.lang.Exception`

---


### getDirectory

```
public java.io.File getDirectory()
```

:   Get the directory that the model(s) will be saved into

    :   **Returns:**: the directory to save to

---


### setDirectory

```
public void setDirectory(java.io.File d)
```

:   Set the directory that the model(s) will be saved into.

    :   **Parameters:**: `d` - the directory to save to

---


### setUseRelativePath

```
public void setUseRelativePath(boolean rp)
```

:   Set whether to use relative paths for the directory.
    I.e. relative to the startup (user.dir) directory

    :   **Parameters:**: `rp` - true if relative paths are to be used

---


### getUseRelativePath

```
public boolean getUseRelativePath()
```

:   Get whether to use relative paths for the directory.
    I.e. relative to the startup (user.dir) directory

    :   **Returns:**: true if relative paths are to be used

---


### getPrefix

```
public java.lang.String getPrefix()
```

:   Get the prefix to prepend to the model file names.

    :   **Returns:**: the prefix to prepend

---


### setPrefix

```
public void setPrefix(java.lang.String p)
```

:   Set the prefix to prepend to the model file names.

    :   **Parameters:**: `p` - the prefix to prepend

---


### globalInfo

```
public java.lang.String globalInfo()
```

:   Global info for this bean. Gets displayed in the GUI.

    :   **Returns:**: information about this bean.

---


### setFileFormat

```
public void setFileFormat(Tag ff)
```

:   Set the file format to use for saving.

    :   **Parameters:**: `ff` - the file format to use

---


### getFileFormat

```
public Tag getFileFormat()
```

:   Get the file format to use for saving.

    :   **Returns:**: the file format to use

---


### validateFileFormat

```
public Tag validateFileFormat(Tag ff)
```

:   Validate the file format. After this bean is deserialized, classes for
    XML serialization may not be in the classpath any more.

    :   **Parameters:**: `ff` - the current file format to validate

---


### setEnvironment

```
public void setEnvironment(Environment env)
```

:   Set environment variables to use.

    :   **Specified by:**: `setEnvironment` in interface `EnvironmentHandler`
    :   **Parameters:**: `env` - the environment variables to use


---


|  |  |  |
| --- | --- | --- |
| **Overview** \| **Package** \| **Class** \| **Tree** \| **Deprecated** \| **Index** \| **Help** \| **Weka's home** | |  |
| **PREV CLASS**   **NEXT CLASS** | **FRAMES**    **NO FRAMES**     **All Classes** |  |
| SUMMARY: NESTED \| FIELD \| CONSTR \| METHOD | DETAIL: FIELD \| CONSTR \| METHOD |  |


---
